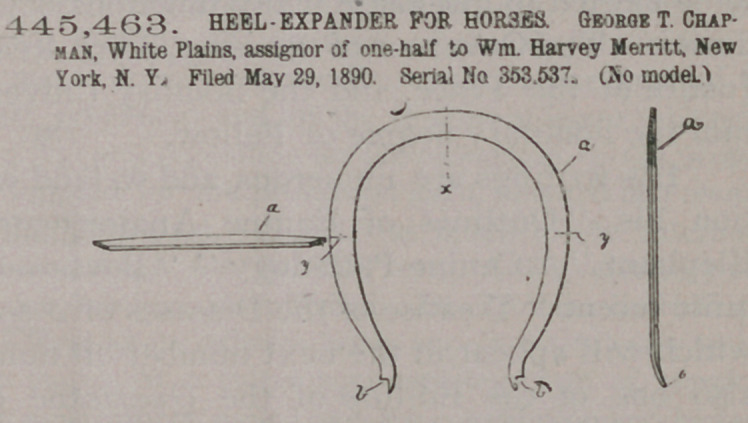# Recent Patents

**Published:** 1891-03

**Authors:** 


### RECENT PATENTS

RELATING TO

### VETERINARY MEDICINE AND ANIMAL INDUSTRY.

*Issued by U. S. Patent Office for Month ending Feb. 17th,* 1891.

445,428. HARNESS. JOHN E. FOSTER, Ferndale, Cal. Filed Sept. 12, 1890. Serial No. 364,770. (No model.)

*Claim.*—1. In a track-harness, and in combination with the saddle, back-strap, and crupper, the hold-back-straps C, secured at their rear ends to the crupper and at their forward ends to the saddle, and the straps D and E, secured at one end to the saddle, and at their opposite ends to the shafts at a point forward and back of the saddle, respectively, substantially as herein described.

2. In a track-harness, and in combination with the saddle, backstrap, and crupper, and at their forward ends to the saddle, the straps D, secured at their rear ends to the saddle and having the sockets secured to their forward ends of the shafts, and the straps E, connecting the saddle with the shafts, substantially as herein described.

3. In a track-harness, and in combination with the saddle, back-strap, and crupper, the holdback-straps C, secured at their rear ends to the crupper and at their forward ends to the saddle, the straps D, secured at their rear ends to the saddle and at their forward ends to the shafts at a point in front of the saddle, and the tightening-straps E, secured at their forward ends to the saddle and at their rear ends to the shafts at a point back of the saddle, substantially as herein described.

4. In a track-harness, and in combination with the saddle, back-strap, and crupper, the holdback-straps C, secured at their rear ends to the crupper and at their forward ends to the saddle, and a strap on each side secured at its ends to the shafts at points forward and back of the plane of the saddle and at its intervening portion to said saddle, substantially as herein described.

5. The track-harness consisting of the saddle with its belly-band, the back-strap with its crupper, the holdback-straps secured to the crupper and to the saddle, the straps secured to the shafts at points forward and back of the plane of the saddle and to said saddle, the neck-strap secured at its lower end to the belly-band, substantially as herein described.

6. In a harness, the combination, with the saddle, of the holdback-straps C and the crupper to which said straps are connected, said crupper having an elongated opening, and a band sliding on the crupper, substantially as herein described.

445,165. CURRY-COMB. LEVI M. DEVORE, Freeport, Ill. Filed Jan. 30, 1890. Serial No. 338,601. (No model.)

*Claim.*—1. In a curry-comb of the class described, the combination, with a flexible back and a loop or handle attached to the one face thereof, of a series of sections fastened to the opposite face thereof in lines approximately parallel to the line of the handle, each of said sections being provided with teeth arranged in curved lines, substantially as and for the purpose set forth.

2. In a curry-comb of the class described, the combination, with a flexible back, of a handle attached to and extending across one face thereof and a series of sections attached to the opposite face of the back in lines approximately parallel to the line of the handle, said sections being provided with teeth arranged in lines made up of a series of reversely-arranged curves, substantially as set forth.

446,380. CRUPPER-FASTENING. GEORGE H. DAVIS, Lacona, N. Y., assignor of one-half to S. H. Barlow, same place. Filed Aug. 4, 1890. Serial No. 360,885. (No model.)

*Claim.*—A crupper-fastening device consisting of the parts A and B, having their edges turned up and corrugated at right angles to the length of the clamp, and provided with an eye 4 in one end thereof, substantially as described, for the purposes as set forth.

445,454. TETHER. FRANCIS M. POWELL and WILLIAM H. VICKERY, Hartwell, Ga. Filed Aug. 29, 1890. Serial No. 363,437. (No model.)

*Claim.*—In a tether, the combination of a stake, a head-block supported thereon so as to turn, and a spring-operated lever fulcrumed in said block, said block being constructed with a front 5, sides 6 6a, an inclined top 7, into which the upper end of a vertical slot in the front 5 extends, and a boss 9, through which extends a perforation at one corner of the block to receive the stake, said vertical slot permitting vertical play of the lever, substantially as set forth.

**445,485. MANE-HOLDER FOR HORSES.** WILLIAM AMBRUSTER, St. Louis, Mo. Filed May 31, 1890. Serial No. 353,695. (No model.)

*Claim.*—1. In a mane-holder for horses, a pair of parallel arms or jaws, a locking device formed on said parallel arms and integrally therewith, and a similar pair of arms hinged to said first-mentioned pair, substantially as described.

2. In a mane-holder for horses, a jaw 2, a U-shaped jaw hinged thereto, its free end extending beyond the end of the first-mentioned jaw forming a loop, whereby it may be secured to the harness, and a collar on the jaw 2 between the ends of the other jaw, substantially as described.

3. In a mane-holder for horses, a jaw 2, a second jaw hinged thereto, an eye on one end of jaw 2, and a hook upon the other end to engage the eye, substantially as described.

4. In a mane-holder for horses, a jaw 2, a second jaw hinged thereto, corrugated plates on each of said jaws, and means whereby said jaws may be fastened together, substantially as described.

5. In a mane-holder for horses, a jaw 2, a second jaw hinged thereto, eace of said jaws having depression, corrugated plates located in said depressions, and elastic material on said corrugated plates.

**445,778. HOOF-EXPANDER.** MICHAEL HEAGERTY, Baltimore Md. Filed Nov. 25, 1890. Serial No. 372,610. (No model.)

*Claim.*—A hoof-expander formed of a single piece of spring metal and comprising a pair of diverging arms *a*, each having a lateral prong *b* at its outer end, two toe bends *c* at the converging ends of the arms, and a U-shaped double back bend *d*, connecting said two toe bends and occupying a position between the said arms *a*, as and for the purpose set forth.

**446,248. MILK TESTING AND SEPARATING MACHINE.** DYER COOPER, Philadelphia, Pa. Filed July 16, 1888. Serial No. 280,111. (No model.)

*Claim.*—1. The combination, in a milk testing or separating machine, of the frame, an open-armed spider mounted on said frame, and mechanism, substantially as described, for driving the spider, said spider having sockets with yielding seats therein, crossheads *e* on the spider, having pockets, glass tubes adapted to the sockets and pockets, and an elastic fastening passing transversely across the tubes, substantially as set forth.

2. The combination of the testing-tubes with the spider having arms with pockets for said tubes and elastic retainers for holding the tubes in the pockets, substantially as described.

**445,920. DEVICE FOR WATERING STOCK.** CHARLES E. BUCKLEY, Amenia Union, N. Y. Filed Oct. 17, 1890. Serial No. 368,384. (No model.)

*Claim*.—1. In a device for watering stock, the combination, with a main reservoir and a distributing-pipe connected therewith, of a series of receptacles connected with the same distributing-pipe, inlet-pipes extending into the receptacles above their bottoms, a cover placed over the upper ends of the inlet-pipes, having a flange depending below the inlets, valves in the inlet-pipes and under the cover, and inlet-openings below the said valves, substantially as specified.

2. In a device for watering stock, the combination, with a main reservoir and a distributing-pipe connected therewith, of a receptacle, an inlet-pipe extending into the receptacle above its bottom, and a cover placed over the upper end of the pipe, having a flange depending below the inlet and inlet-passages, combined for the purpose described.

3. In a device for watering stock, the combination, with a main reservoir and a distributing-pipe connected therewith, of a receptacle, an inlet-pipe extending into and above the bottom of the receptacle having longitudinal grooves, and a cover having a depending flange and which is placed over the inlet-pipe, substantially as shown.

**446,067. CONTROLLING-GEAR FOR DRAFT-HORSES.** ROBERT S. KINKEAD, Lexington, Ky., Filed Nov. 16, 1889. Serial No. 330,566. (No model.)

*Claim*.—1. A controlling-gear consisting of an auxiliary bit, an independent rein attached thereto, nose and head guides for said rein, and a pivoted lever on the vehical having a friction-pulley around which the independent rein runs before it is attached to the wagon body, substantially as described.

2. In a controlling-gear of the character described, the combination of a checkrein, a water-hook ring, a button thereon over which the checkrein is engaged, a friction-roller engaged in the bight of the checkrein behind the water-hook ring, and a connecting-rein engaged with the roller and extending back to the vehicle, substantially as described.

3. In a controlling-gear of the character described, the combination of a slotted plate adapted to be attached to the bottom of the vehicle, a guide-roll mounted thereon, a lever fulcrumed upon said plate and operating through the slot, a pivoted roll carried at the end of the lever, and a connecting-rein secured at one end to said plate and passing thence around said roll at the end of the lever and forward ultimately to and connected with a bit in the mouth of the animal, substantially as set forth.

445,463. HEEL-EXPANDER FOR HORSES. GEORGE T. CHAPMAN, White Plains, assignor of one-half to Wm. Harvey Merritt, New York, N. Y. Filed May 29, 1890. Serial No. 353,537. (No model.)

*Claim.*—1. The lyre-shaped heel-expanding spring consisting of the thin flat bar widest at the middle and gradually tapered to the ends and having the pitch in the cross-section of the bar oblique to the plane of the entire spring, substantially as described.

2. The lyre-shaped heel-expanding spring consisting of the thin flat bar widest at the middle and gradually tapered to and upturned and outwardly projecting at the ends and having the pitch of the cross section of the bar oblique to the plane of the entire spring, substantially as described.

## Figures and Tables

**Figure f1:**
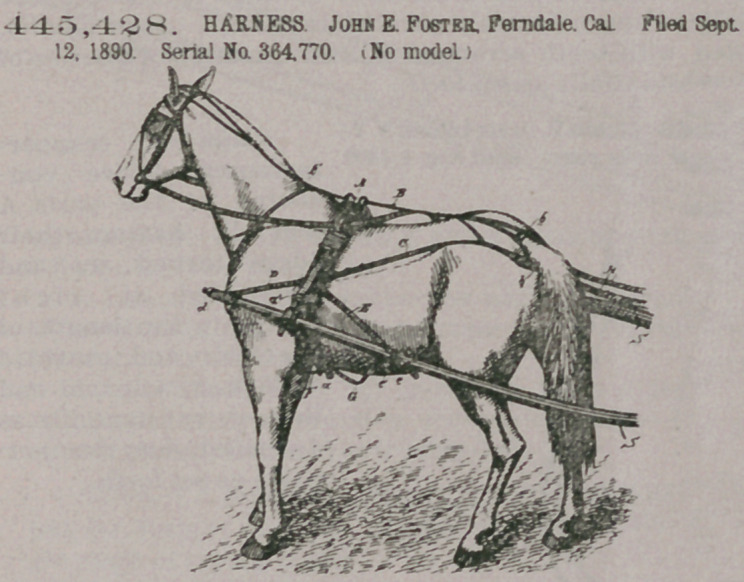


**Figure f2:**
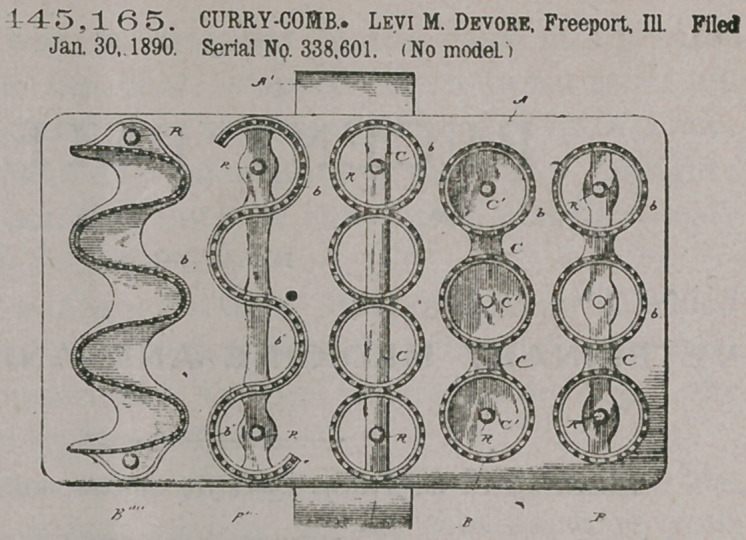


**Figure f3:**
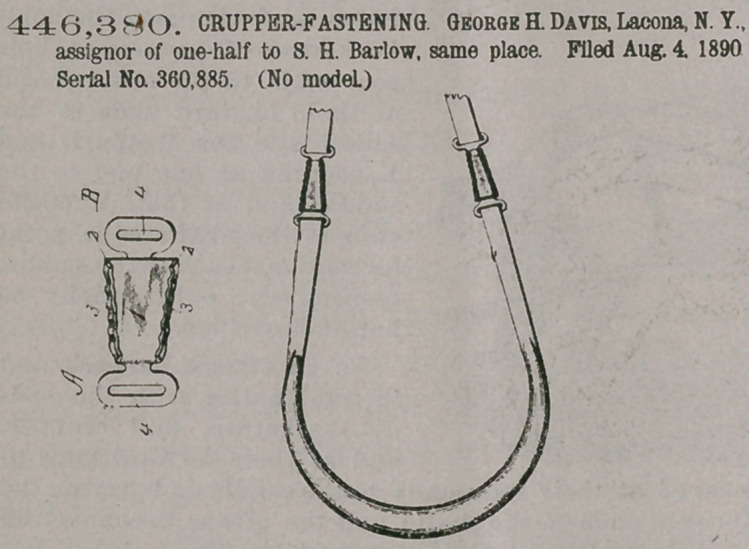


**Figure f4:**
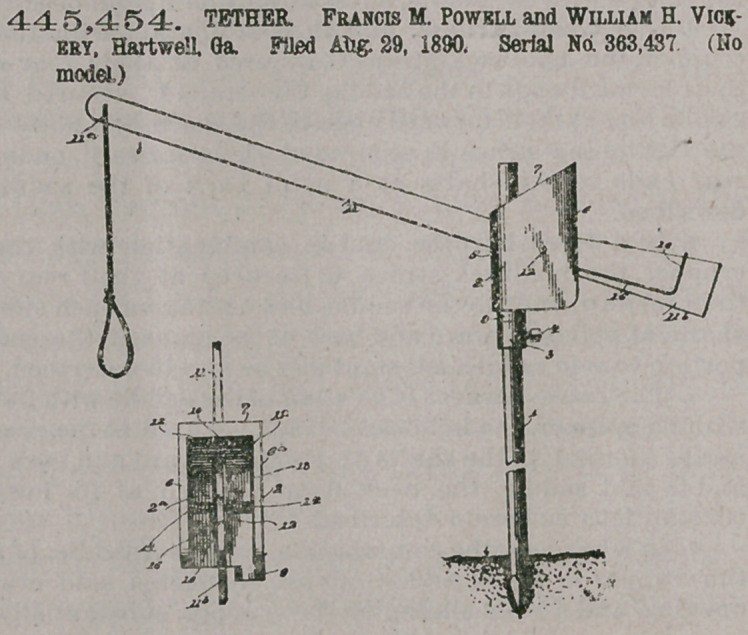


**Figure f5:**
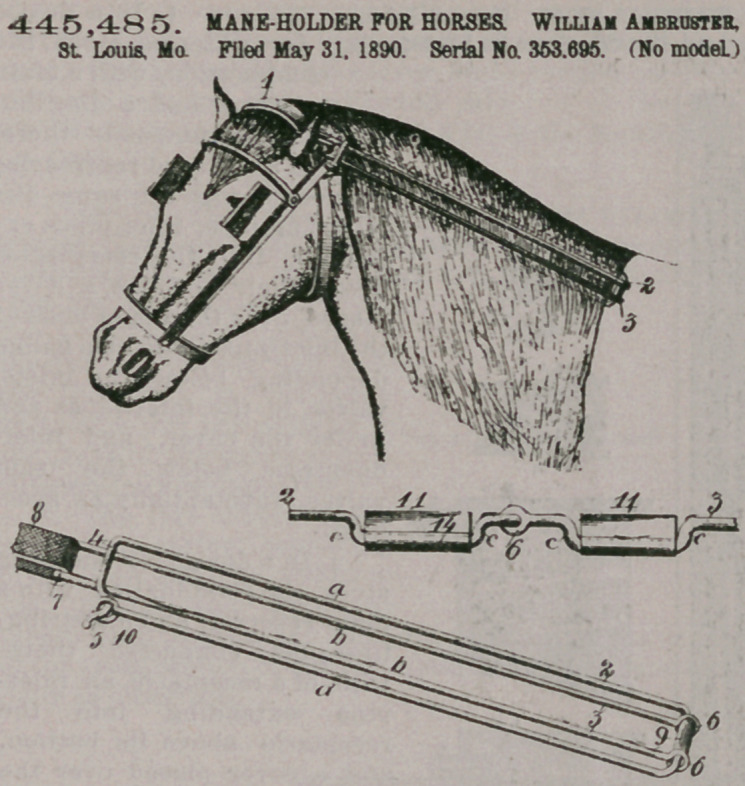


**Figure f6:**
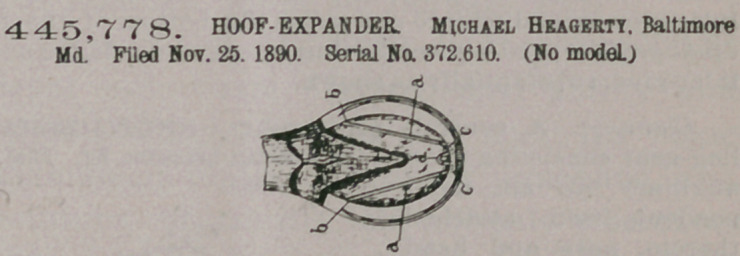


**Figure f7:**
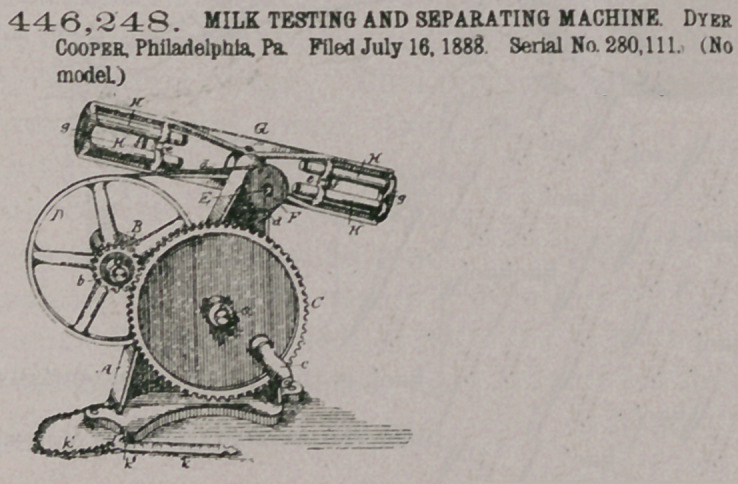


**Figure f8:**
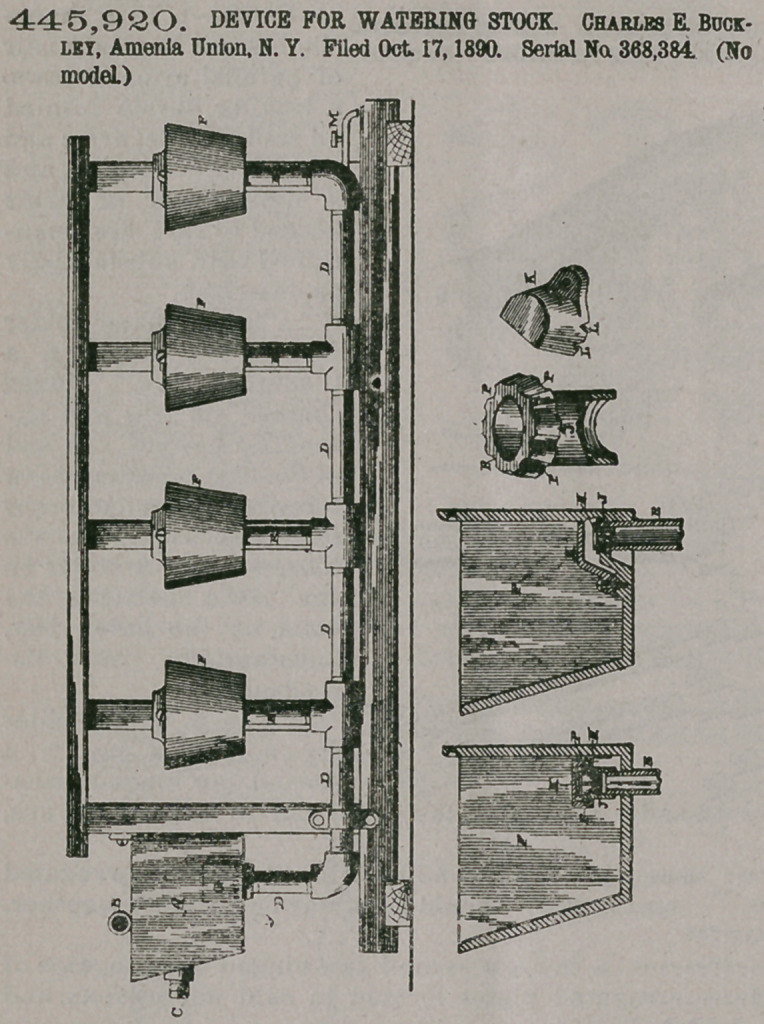


**Figure f9:**
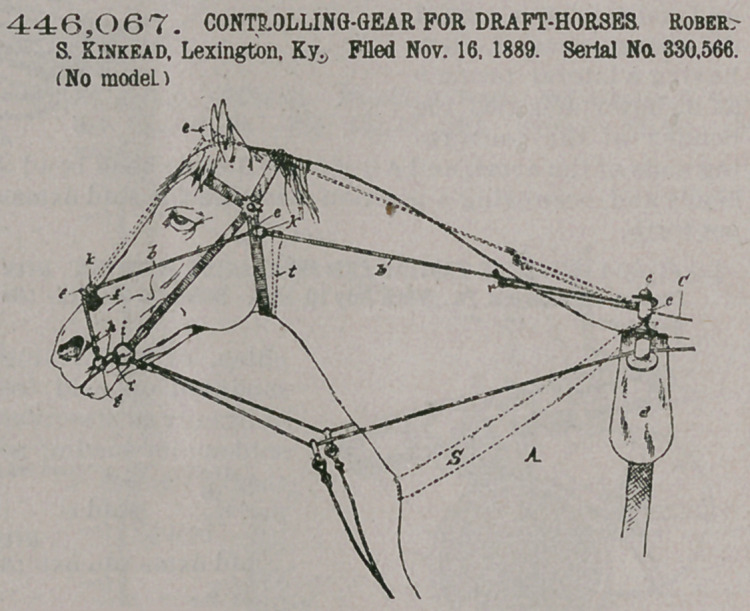


**Figure f10:**